# Laminar-dependent effects of cortical state on auditory cortical spontaneous activity

**DOI:** 10.3389/fncir.2012.00109

**Published:** 2012-12-21

**Authors:** Shuzo Sakata, Kenneth D. Harris

**Affiliations:** ^1^Center for Molecular and Behavioral Neuroscience, Rutgers, The State University of New JerseyNewark, NJ, USA; ^2^Strathclyde Institute of Pharmacy and Biomedical Sciences, University of StrathclydeGlasgow, UK; ^3^Centre for Neuroscience, University of StrathclydeGlasgow, UK; ^4^Department of Bioengineering, Electrical and Electronic Engineering, Imperial CollegeLondon, UK

**Keywords:** sensory cortex, cell-type, cortical circuit, ensemble recording, slow oscillation

## Abstract

Cortical circuits spontaneously generate coordinated activity even in the absence of external inputs. The character of this activity depends on cortical state. We investigated how state affects the organization of spontaneous activity across layers of rat auditory cortex *in vivo*, using juxtacellular recording of morphologically identified neurons and large-scale electrophysiological recordings. Superficial pyramidal cells (PCs) and putative fast-spiking interneurons (FSs) were consistently suppressed during cortical desynchronization. PCs in deep layers showed heterogeneous responses to desynchronization, with some cells showing increased rates, typically large tufted PCs of high baseline firing rate, but not FSs. Consistent results were found between desynchronization occurring spontaneously in unanesthetized animals, and desynchronization evoked by electrical stimulation of the pedunculopontine tegmental (PPT) nucleus under urethane anesthesia. We hypothesize that reduction in superficial layer firing may enhance the brain's extraction of behaviorally relevant signals from noisy brain activity.

## Introduction

The cortex is never silent, but produces complex spontaneous population firing patterns even in the absence of sensory input. The structure of cortical spontaneous activity varies from moment to moment, with the different activity patterns referred to as different cortical states (Steriade, 2001; Castro-Alamancos, 2004; Poulet and Petersen, 2008; Curto et al., 2009; Haider and McCormick, 2009; Harris and Thiele, 2011). During slow wave sleep and under most anesthetic conditions, the cortex exhibits a “synchronized” state, where neural population activity fluctuates between phases of generalized spiking and silence, corresponding to phases of intracellular depolarization and hyperpolarization known as up and down states (Steriade et al., 1993b; Hoffman et al., 2007; Crunelli and Hughes, 2010; Harris and Thiele, 2011). In active wakefulness, these fluctuations are replaced by tonic activity characteristic of a “desynchronized” state (Castro-Alamancos, 2004; Poulet and Petersen, 2008; Renart et al., 2010; Harris and Thiele, 2011). Nevertheless, the details of how state affects patterns of cortical population activity are not yet fully clear, for example, with regards to how state changes affect the activity of different cortical cell classes and layers (Chen et al., 2008; De Kock and Sakmann, 2009; Gentet et al., 2010; Hirata and Castro-Alamancos, 2010; Niell and Stryker, 2010; Constantinople and Bruno, 2011; Ushimaru et al., 2012).

Different cortical cell classes show differential patterns of both spontaneous and evoked firing. In auditory and somatosensory cortex, both spontaneous and evoked activity are very sparse in layer (L) 2/3 pyramidal cells (PCs), whereas fast-spiking interneurons (FSs) of all layers and large L5 PCs show denser activity patterns (De Kock et al., 2007; Wallace and Palmer, 2008; Sakata and Harris, 2009). The way cortical state affects the firing of different cell classes has been addressed by studies in visual and somatosensory cortex: for example, superficial FS cells in the barrel cortex are suppressed during desynchronization seen during whisking (Gentet et al., 2010) whereas they become more active in thalamic recipient layers during induced desynchronization in anesthetized animals (Hirata and Castro-Alamancos, 2010) as well as in superficial layers of the visual cortex during running, a state that is expected to cause desynchronization as evidenced by decreased low-frequency power (Niell and Stryker, 2010; Harris and Thiele, 2011).

Although cortical state clearly affects auditory information processing (Hubel et al., 1959; Edeline et al., 2001; Issa and Wang, 2008; Lakatos et al., 2008; Curto et al., 2009; Otazu et al., 2009; Harris and Thiele, 2011; Harris et al., 2011; Marguet and Harris, 2011), little information is available about state-dependent and cell-type-specific firing in the auditory cortex. Here we investigate how cortical state affects the laminar structure of spontaneous activity in rat primary auditory cortex, using juxtacellular recording of single morphologically identified neurons, and parallel extracellular recording of neuronal populations. We report that in both urethane-anesthetized and unanesthetized animals, putative FS cells and superficial layer PCs reduce their spiking during cortical desynchronization neurons of other PC classes exhibit diverse effects.

## Materials and methods

### Animals

We used fifty-nine adult Spargue-Dawley rats (range 200–517 g, both sexes) and seven adults (range 270–380 g, male) for the anesthetized and unanesthetized experiments, respectively. All procedures were approved by the Institutional Animal Care and Use Committee of Rutgers University. Detailed experimental procedures were described elsewhere (Curto et al., 2009; Sakata and Harris, 2009).

### Anesthetized experiments

#### Surgical procedures

Animals were anesthetized with 1.5 g/kg urethane. An additional solution was administered to reduce brain edema (dexamethasone, 0.10 mg). Lidocaine (2%, 0.10–0.20 mg) was also administered subcutaneously at the site of incision. Additional doses of urethane (~0.2 g/kg) were given if necessary. The animal was placed in a custom naso-orbital restraint that left the ears free and clear. Body temperature was retained at 37°C with a feedback temperature controller (40-90-8C, FHC). After reflecting the temporalis muscle, left auditory cortex was exposed via craniotomy and a small duratomy was carefully performed. During recording, the cavity was filled with 1–1.5% agar/0.1 M phosphate buffered saline (PBS) to reduce pulsation.

#### Electrophysiology

Neuronal activity in the auditory cortex was recorded simultaneously with a 16 or 32 channel “silicon probe” (NeuroNexus Technologies), and a glass electrode for recording individual morphologically identified neurons. All recordings were performed in a single-walled soundproof box (MAC3, IAC). For histological verification of probe tracks, the rear of probes was painted with DiI (Invitrogen, D-282, ~10% in ethanol). Pipettes were pulled from glass capillaries (World Precision Instruments) using a vertical puller (Narishige, PE-2 or PC-10) and the pipette tip was broken under a microscope. Pipettes were filled with 1.5–2.0% Neurobiotin (Vectastain) dissolved in 0.5 M NaCl. Their resistance was 10–20 MΩ in the agar on the cortical surface. Broadband signals (>1 Hz) from the silicon probe were amplified (1000x) (PBX2, Plexon), and narrow-band signals (100–3 kHz) from the pipette were amplified (1000x) (MultiClamp 700B, Molecular Devices). All data were digitized at 20 kHz and stored for offline analysis. Recording was followed by juxtacellular labeling (Pinault, 1996): positive current pulses (0.5–8 nA, 50% duty cycle) were applied at 2 or 5 Hz. The current was slowly increased until it drove the discharge activity. After that, the current was immediately adjusted to 0.5–2 nA and then this rhythmic activity was maintained for up to 20 min. After a survival period (30 min to 6 h), histological procedures were conducted (see below).

#### PPT stimulation

The bone above the pedunculopontine tegmental (PPT) nucleus was removed, and a concentric bipolar stimulation electrode (20–50 kΩ at 1 kHz, SNE-100; David Kopf Instruments) was implanted into the PPT (7.5 mm posterior from the bregma, 1.8 mm lateral from the midline, 6.5–7.0 mm deep from the dorsal surface of the brain). A 1 s pulse train (100 Hz, 200 μs duration, 50–100 μA) was applied to induce the desynchronized state. After the experiment, we verified the location of stimulating electrode tips histologically.

### Unanesthetized experiments

In initial surgery, animals were anesthetized by ketamine (100 mg/kg) and xylaxine (10 mg/kg), and placed in a stereotaxic apparatus (David Kopf Instruments). A head-post (Thorlabs, Inc.) was attached with dental cement (3M ESPE, RelyX Luting Cement), and the left temporal muscle removed and covered with biocompatible glue and dental cement. After a recovery period (>48 h), animals were lightly water-deprived, and handling (5–10 min/day) and head-fixation training began. Training was performed for at least 5 sessions, during which the duration of restraint was gradually extended. Ten percent sucrose was frequently given during training and water was freely available for at least 1 h after daily training. On the day of recording, craniotomy and duratomy were carefully performed under anesthesia (0.8–5% isoflurane). Neither skin nor muscle was cut during this surgery. After a short recovery period (>1 h), recording began. Only silicon probe recording was conducted. During recording, animals were video-monitored. If animals showed discomfort, experiments were immediately terminated. Recording sessions usually lasted 1–2 h. All training and recordings were performed in the soundproof box.

### Histology

#### Histological procedures

After electrophysiological experiments, rats were deeply anesthetized and perfused transcardially with physiological saline followed by 4% paraformaldehyde plus 0.5% glutaraldehyde/0.1 M phosphate buffer, pH 7.4. After a 12–14 h postfixation in the same fixative without glutaraldehyde, brains were cut into 80 μm coronal sections with a microtome (VT1000S, Leica), and the sections collected and placed in 0.1 M PBS. For verification of silicon probe tracks, the free-floating sections were counterstained with NeuroTrace (1:80; Invitrogen) in PBS for 20 min at room temperature. For visualization of juxtacellularly labeled cells, the free-floating sections were incubated in PBS containing 0.3% H_2_O_2_ for 20 min at room temperature, then processed with an avidin-biotinylated horseradish peroxidase complex (1:100; Vectastain ABC Elite kit) in PBS with 0.2% Triton X-100 at room temperature for 2.5 h or at 4°C overnight. The reaction was visualized with nickel-enhanced coloring solution (0.2 mg/mL daminobenzidine, 0.03% H_2_O_2_, 0.03% nickel chloride in tris-buffered saline). The sections were mounted on gelatin-coated slides, dehydrated, and embedded in DPX Mountant (Fisher Scientific). Selected sections were also counterstained with cresyl violet or thionin to determine cortical layers.

#### Histological analysis

Juxtacellularly labeled cells were identified morphologically as PCs by pyramidal shape soma and a prominent apical dendrite. Laminar borders were determined based on background staining and/or Nissl counterstain under microscope. To quantify morphological features, only the peri-somatic region was reconstructed and analyzed with Neurolucida (MicroBrightField, Inc.). Dendritic diameter was defined as the apical dendrite diameter 10 μm from the center of somatic contour. L5 thick PCs were defined as a L5 PC with a thicker (>2.5 μm of diameter) apical dendrite. As shown previously (Figure S1A in Sakata and Harris, 2009), we observed a cluster of L5PCs with <2.5 μm of diameter.

### Data analysis

#### Spike detection and sorting

All spike detection and sorting took place off-line. Multiunit activity (MUA) was defined as spiking activity without spike sorting. For spike sorting, freely available software was used (KlustaKwik, http://klustakwik.sourceforge.net; Klusters, http://klusters.sourceforge.net). Unit isolation quality was assessed by “isolation distance” (Harris et al., 2001; Schmitzer-Torbert et al., 2005); only cells with values ≥20 were further analyzed as single-units.

#### Single-unit classification

We classified spike-sorted units based on three features of average spike waveforms: trough to peak time, half amplitude width, and the asymmetry index of peak amplitude (i.e., ratio of the difference between right and left baseline-to-peak amplitudes and their sum). We performed k-means clustering in this three-dimensional space to separate into two classes. Putative FSs were characterized by narrower spike waveforms and relatively higher asymmetry index. Putative PCs were characterized by wider spike waveforms and relatively smaller asymmetry index (see also Figure S4 in Sakata and Harris, 2009). Spontaneous firing rates under anesthesia were 0.9 ± 0.2, 3.0 ± 0.2, 5.8 ± 2.1, and 5.1 ± 0.8 Hz for superficial PCs, deep PCs, superficial FSs, and deep FSs, respectively, whereas those in the unanesthetized condition were 2.2 ± 0.5, 4.5 ± 0.4, 7.5 ± 4.4, and 13.5 ± 3.5 Hz for superficial PCs, deep PCs, superficial FSs, and deep FSs, respectively. It is important to note that in primates, a subset of PCs exhibit narrow spikes (Vigneswaran et al., 2011). However, our conclusions based on PC identification from spike width were also confirmed in recordings of morphologically identified PCs under anesthesia.

We further classified spike-sorted units based on the depth of spike-sorted units, which was estimated from the stereotaxically estimated depth of the electrode tip and spike waveform profiles (Sakata and Harris, 2009). Somatic location was estimated as the recording site with maximum peak-to-trough amplitude based on mean spike waveforms. Putative superficial layers (L2/3) and deep layers (L5) were defined as 0–500 μm and 800–1100 μm from surface, respectively. Note that to reduce the risk of misclassification (such as might arise from somatodendritic backpropagation of action potentials in large PCs), this criterion was deliberately conservative, with cells of intermediate depth or deeper than putative L5 not assigned to either group for further analysis. Correcting for shrinkage of sections with an assumption that cortical thickness (from pia to the bottom of L6) was approximately 1600 μm (Paxinos and Watson, 1998), we found that morphologically identified L5 PCs were located at a depth of 695–1100 μm.

#### State evaluation

In experiments where desynchronization was evoked by PPT stimulation under anesthesia, we verified that the stimulation was effective by computing total spectral power of local-field potential (LFP) at 0–7 Hz. Out of 37 morphologically identified PCs, for 34 we found sufficient stimulation-induced desynchronization to allow further analysis: 7 L2/3 PCs, 7 L4 PCs, 11 L5 slender PCs (apical dendrite diameter <2.5 μm), 4 L5 thick PCs (apical dendrite diameter >2.5 μm), and 5 L6 PCs. Out of four indentified non-PCs (probably GABAergic interneurons), we found sufficient stimulation-induced desynchronization for three cells. For spike-sorted single units, six of 37 datasets were excluded due to the failure of PPT stimulations. This left a database of 401 single units, of which 45 were classified as putative superficial PCs, 303 as putative deep PCs, 10 as putative superficial FSs, and 43 as putative deep FSs. Firing rates in synchronized and desynchronized states were estimated from 2-s windows immediately before and after stimulations, respectively. To assess statistical significance of firing rate changes by induced desynchronization, we performed pairted *t*-test.

**Figure 1:**
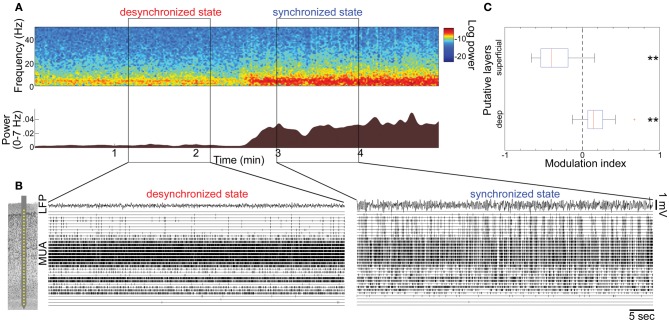
**State-dependent spontaneous activity across cortical layers under anesthesia. (A)** Spontaneous shift of cortical states. The spectrogram (*top*) was computed from local-field potentials (LFPs) in the auditory cortex without auditory stimuli. Log-power was color-coded. The bottom panel shows a history of total LFP power at 0–7 Hz. **(B)** Multiunit activity (MUA) across cortical layers and LFPs during the desynchronized and synchronized state, with a schematic drawing of a 32 site linear electrode. LFP was taken from channel 16, likely located in infragranular layers. **(C)** Modulation index of spontaneous population activity in putative L2/3 (0–500 μm from cortical surface) and L5 (800–1100 μm). ^**^p < 0.005 (*t*-test).

To evaluate spontaneous shifts of brain states in both anesthetized and unanesthetized conditions, we considered two parameters: LFP power at 0–7 Hz and appearance of global spiking and silent phases, where presumptive up and down states were recognized based on MUA. The desynchronized state was characterized by lower spectral power and no clear transition between up and down states (Figure 1). LFP was measured across all channels. While LFP for this analysis was taken primarily from infragranular layers, the spontaneous shifts of brain states could be confirmed in firing rates of all layers. We found sufficient spontaneous state shifts for further analysis in 21 and 6 datasets of anesthetized and unanesthetized experiments, respectively. For spike-sorted single units in unanesthetized experiments, 39 were classified as putative superficial PCs, 115 as putative deep PCs, and 17 putative FSs (4 and 13 in superficial and deep layers, respectively). To assess statistical significance of firing rate changes by cortical states, we performed unpaired *t*-test as cortical states were evaluated at 1-s resolution.

#### Modulation index

For comparison of firing rates between the synchronized and desynchronized states, we computed the modulation index as follows: (FR_*d*_ − FR_*s*_)/(FR_*d*_ + FR_*s*_), where FR_*d*_ and FR_*s*_ are firing rates in the desynchronized and synchronized states, respectivsely.

## Results

### State-dependent spontaneous population activity across layers in anesthetized animals

To investigate how global brain states are associated with spiking activity at the local circuit level, we began by monitoring spontaneous population activity across cortical layers in the primary auditory cortex (A1) of urethane-anesthetized rats (Figure 1). Consistent with previous reports (Clement et al., 2008), cortical activity frequently and spontaneously changed between synchronized and desynchronized states (typical duration of synchronized state epochs, 167 ± 10 s; desynchronized state, 165 ± 17 s, mean ± SEM). During the synchronized state, low frequency LFP power was increased (Figure 1A), and MUA appeared synchronously across cortical layers (Figure 1B), consistent with an alternation of phases of generalized spiking and silence (up and down states) as previously described (Sakata and Harris, 2009). During the desynchronized state, the silent phase disappeared and neural population activity was tonically active in the deep layers. However, we noticed that desynchronization was associated with a powerful decrease in superficial layer activity compared with the synchronized state (Figure 1B). Data from 21 penetrations confirmed that MUA firing rates significantly decreased in superficial layers, but increased in deep layers during desynchronization under urethane anesthesia (Figure 1C).

### State-dependent and cell-type-specific spontaneous firing in anesthetized animals

**Figure 2:**
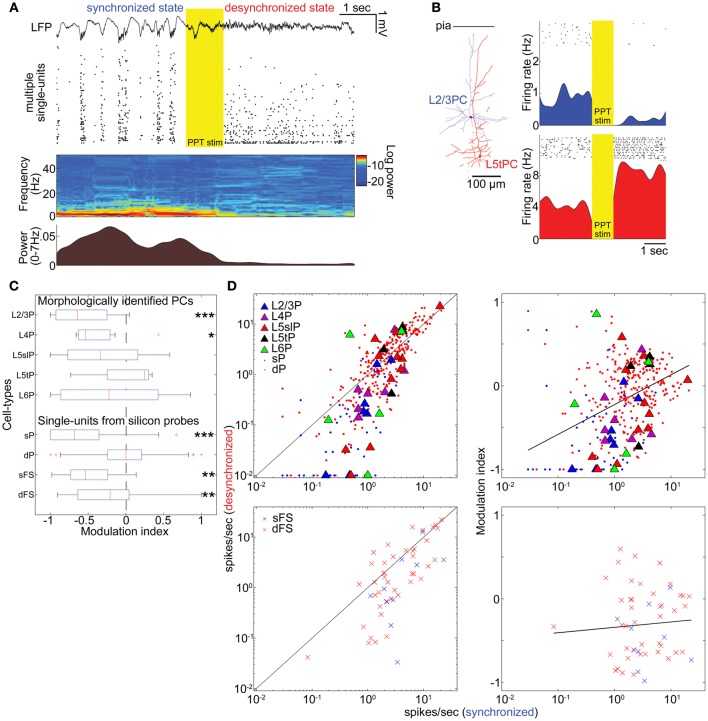
**State-dependent and cell-type-specific spontaneous firing. (A)** An example of pedunculopontine tegmental (PPT) nucleus stimulation and neural population activity in the auditory cortex. LFP, multiple single-units, spectrogram, and a history of low-frequency LFP power are shown. **(B)** Examples of morphologically identified pyramidal cells (PCs) and the effects of PPT stimulation on their spiking. Rasters show response of these cells to repeated PPT stimuli; lower graphs show peri-stimulus time histograms. **(C)** Modulation index of morphologically identified PCs and extracellularly classified single-units. L5slP, L5 slender PCs; L5tP, L5 thick PCs; sP and dP putative superficial and deep PCs, respectively; sFS and dFS, putative superficial and deep fast-spiking interneurons (FSs), respectively. ^*^p < 0.05, ^**^p < 0.01, and ^***^p < 0.005 (signed-rank test). **(D)** Mean firing rate during the synchronized state plotted against that during the desynchronized state (*left column*) and modulation index (*right column*) for morphologically identified and putative PCs (*top row*), and putative FSs (*bottom row*). Solid lines in left panels correspond to equality; those in right panels are linear regression fits. Please note that linear fitting was done with a semi-logarithmic scale.

To systematically assess how cortical state affects the firing of individual neurons, we first used electrical stimulation of the PPT to induce the desynchronized state repetitively in anesthetized animals (Figure 2A), a long-standing paradigm for manipulation cortical states under anesthesia (Steriade et al., 1991, 1993a; Rudolph et al., 2005; Curto et al., 2009). Neuronal populations were recorded extracellularly using silicon probes, simultaneously with juxtacellular recording to identify individual recorded neurons morphologically (Sakata and Harris, 2009). This combination allowed us to systematically assess state-dependent and cell-type specific spontaneous firing *in vivo*.

The effects of desynchronization on single-cell firing varied between neurons. Figure 2B shows two representative examples of morphologically identified PCs. In a L2/3 PC, sparse activity during the synchronized state was further reduced by PPT stimulation. In a L5 thick PC, spiking activity became faster after PPT stimulation. Figure 2C summarizes how the firing of different cell types was modulated by cortical state by calculating a modulation index defined as (FR_*d*_ − FR_*s*_)/(FR_*d*_ + FR_*s*_), where FR_*d*_ and FR_*s*_ are firing rates in the desynchronized and synchronized states. The number of cells significantly modulated was assessed by statistically comparing this index to 0 (Wilcoxon signed-rank test). As summarized in Table 1, half of morphologically identified L2/3 PCs (4 out of 7) were significantly suppressed during desynchronization, while none were enhanced. L4 PCs were also generally suppressed (5 out of 7 suppressed, 1 enhanced). The effect of desynchronization on PCs in infragranular layers was heterogeneous, even within anatomically defined classes: L5 slender PCs were suppressed or enhanced (5 and 3 cells of 11), as were L5 thick PCs (2 and 1 cells out of 4) and L6 PCs (2 and 2 cells out of 5). Out of three non-PCs (presumably GABAergic neurons), two were significantly suppressed.

**Table 1:** **Summary of state-dependent and cell-type-specific spontaneous firing**.

**Cell-type**	**Number of cells**	**Enhanced:Suppressed**
**ANESTHETIZED CONDITION**		
L2/3PCs	7	0:4
L4PCs	7	1:5
L5slPCs	11	3:5
L5tPCs	4	2:1
L6PCs	5	2:2
sPCs	45	0:23
dPCs	303	86:68
sFSs	10	0:7
dFSs	43	7:19
**UNANESTHETIZED CONDITION**		
sPCs	39	8:23
dPCs	115	31:47
sFSs	4	1:3
dFSs	13	2:7

Cell-types, the numbers of analyzed neurons, and the numbers of neurons which were significantly modulated by cortical states were listed. Please note that “enhanced” and “suppressed” mean firing changes relative to the synchronized state. PCs, pyramidal cells; FSs, fast-spiking interneurons; L5slPCs, L5 slender PCs; L5tPCs, L5 thick PCs; sPCs and dPCs, superficial and deep PCs, respectively; sFSs and dFSs, superficial and deep FSs, respectively.

To confirm these results on a larger data set, we analyzed spike-sorted single units recorded simultaneously using silicon probes. Putative FSs were identified by spike waveforms (Sakata and Harris, 2009). Consistent with the juxtacellular recordings, we found that superficial firing rates were reduced during desynchronized state regardless of cell types (23/45 putative PCs, 7/10 putative FSs suppressed, none enhanced), whereas deep-layer cells showed a mixed pattern, with putative PCs somewhat more likely to show enhancement than suppression (86 vs. 68 out of 303 cells), and putative FSs more likely to show suppression (19 vs. 7 out of 43 cells) (Figures 2C,D and Table 1). We found that the propensity of a cell to show enhanced firing under desynchronization was stronger for neurons of higher baseline firing rate within the population of PCs (putative PCs + identified PCs, *r* = 0.32, *p* < 0.0001), but not within putative FSs (*r* = 0.05, *p* = 0.72) (Figure 2D).

These effects of PPT stimulation: near-universal suppression of activity in the superficial layers, and mixed effects in the deep layers with PCs showing slight net enhancement, were consistent with the observations in Figure 1, where desynchronization occurred spontaneously. Furthermore, they indicate that the weak effect of desynchronization on deep layer MUA does not mean a lack of modulation, but rather a cancellation of enhancement and suppression in different populations.

**Figure 3:**
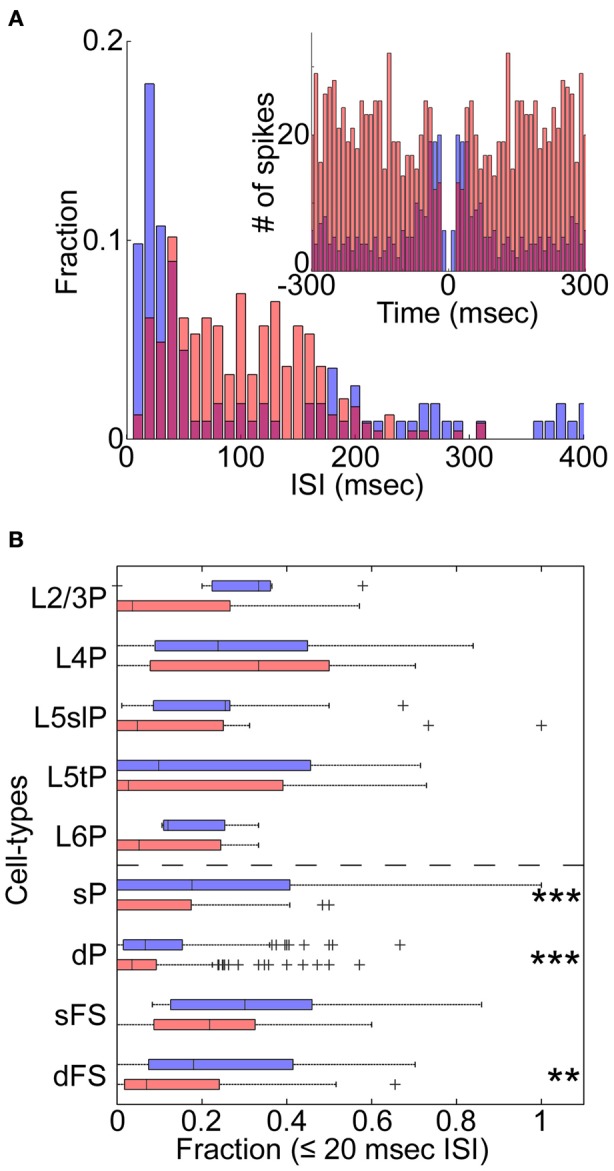
**State-dependent burst firing. (A)** An example of the distribution of inter-spike intervals (ISIs) for a morphologically identified L5tPC (see also Figure 2B) during the synchronized (blue) and desynchronized states (red). Inset: auto-correlogram. **(B)** Fraction of burst-like firing (≤20 ms ISIs) across cell-types during the synchronized (blue) and desynchronized states (red). ^**^p < 0.005 and ^***^p < 0.0005 (Kruskal–Wallis test with *post-hoc* rank sum test).

We next asked whether changes in cortical state were accompanied by changes in spiking patterns other than spike rates (De Kock and Sakmann, 2008). The example L5tPC shown in Figure 2B not only increased its firing rate during the desynchronized state but also changed its firing behavior from bursting during the synchronized state to regular spiking during the desynchronized state (Figure 3A). Across the recorded population, putative deep PCs significantly decreased the fraction of burst-like activity (≤20 ms inter-spike intervals) during the desynchronized state (Figure 3B). A similar trend was observed in all neuronal classes, also reaching statistical significance in putative superficial PCs and deep FSs (Figure 3B) (similar results were also obtained with 10, 15, and 25 ms time windows). Thus, different cortical states were characterized not just by cell-type-specific firing rate, but also by distinct spiking patterns.

### State-dependent and cell-type-specific spontaneous firing in unanesthetized animals

**Figure 4:**
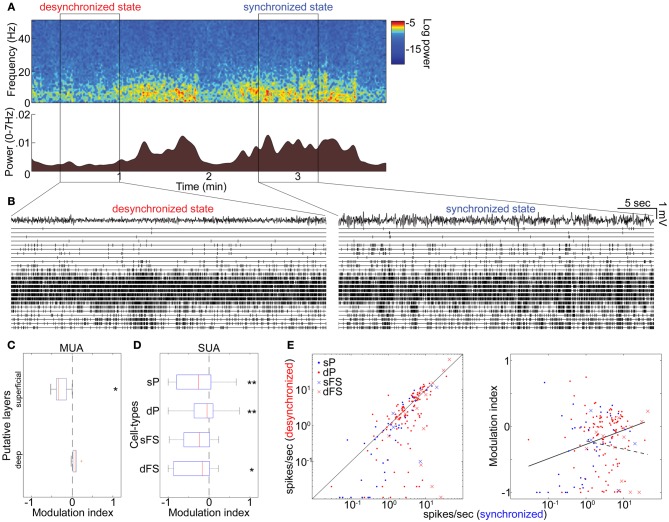
**State-dependent and cell-type-specific spontaneous firing in unanesthetized animals. (A)** Spontaneous shift of cortical states. The spectrogram (*top*) was computed from LFPs in the auditory cortex without auditory stimulation. The bottom panel indicates a history of total LFP power at 0–7 Hz. **(B)** MUA across layers and LFPs in the desynchronized (*left*) and synchronized states (*right*). **(C)** Modulation index of spontaneous population activity in putative L2/3 (0–500 μm from cortical surface) and L5 (800–1100 μm). ^*^p < 0.05 (*t*-test). **(D)** Modulation index of extracellularly classified single-unit activity (SUA) in putative L2/3 and L5. ^*^p < 0.05 and ^**^p < 0.005 (signed-rank test). **(E)** Firing rate during the synchronized state plotted against that during the desynchronized state (*left*) and modulation index (*right*) for putative PCs and putative FSs. Solid line in left panel corresponds to equality; that in right panel is linear regression fit for putative PCs. Dotted line in right panel is linear fit for putative FSs. Linear fitting was done with a semi-logarithmic scale.

To investigate whether these results can be generalized to drug-free animals, we analyzed data collected from head-restrained, unanesthetized rats. As before, we began by examining the structure of MUA across cortical layers simultaneously (Figures 4A,B). Although spontaneous firing rates were generally higher compared to those under anesthetized conditions (Figure S14 in Sakata and Harris, 2009), an alternation between synchronized and desynchronized states was again observed (Figure 4A) (typical duration of synchronized epochs state: 197 ± 59 s, desynchronized state: 423 ± 157 s, mean ± SEM). Superficial multiunit firing was reduced during desynchronization (4 out of 5 data sets), with deep-layer MUA showing little effect (Figure 4C). Examination of spike-sorted single-unit activity (Figures 4D,E and Table 1) confirmed that firing rates were generally suppressed during desynchronization in putative superficial PCs (23 vs. 8 out of 39 cells) and putative FSs of both superficial and deep layers (10 vs. 3 out of 17 cells). The medians of modulation index for combination of superficial and deep FSs were significantly lower than 0 (*p* < 0.05, signed-rank test). On the other hand, deep putative PCs showed a mixture enhancement and suppression (31 and 47 out of 115 cells), this time showing a tendency toward suppression rather than enhancement. The fraction of high frequency spiking activity (≤20 ms ISIs) was apparently decreased only in putative deep PCs during the desynchronized state (0.16 ± 0.01 and 0.12 ± 0.01 in the synchronized and desynchronized states, respectively) (*p* = 0.11, rank sum test). As in the anesthetized data, the effect of desynchronization on a cell was correlated with the cell's baseline firing in the synchronized state, but this effect was only found in putative PCs (putative PCs, *r* = 0.21, *p* < 0.01; putative FSs, *r* = −0.14, *p* = 0.60) (Figure 4E). These results indicate that the effects on firing rate of spontaneous desynchronization in unanesthetized animals are similar to those of spontaneous and PPT-evoked desynchronization under urethane.

## Discussion

We investigated the effects of cortical desynchronization on spontaneous firing rates in identified neural populations of auditory cortex. Both PCs in superficial layers and putative FSs reduced their activity during desynchronized state, but diverse effects were seen in PCs of other layers. The ability of desynchronization to enhance neuronal spiking was positively correlated with a baseline firing rate of PCs, but not FSs. Results were generally consistent for different types of desynchronization (PPT-evoked and spontaneous desynchronization under urethane anesthesia, spontaneous desynchronization in unanesthetized animals).

Superficial layer PCs are sparsely active (Sakata and Harris, 2009). Their decreased firing rate during the desynchronized state is in a sense surprising: whereas during the synchronized state neurons can only fire during up states, this constraint is removed by desynchronization. This observation, together with the diverse rate changes observed in other cell-types, suggests that the effects of cortical state on mean spontaneous firing rate is not simply due to a change in the total amount of “up state time,” but reflects a true state-dependent modulation of excitability that differs between cell classes.

A number of studies have investigated the effects of cortical state on auditory cortical function (Edeline et al., 2001; Issa and Wang, 2008; Lakatos et al., 2008; Curto et al., 2009; Otazu et al., 2009; Harris and Thiele, 2011; Marguet and Harris, 2011), though to our knowledge none have yet identified its effects on different morphological cell classes. This issue has however been addressed in other modalities. In the somatosensory barrel cortex, active whisking, a behavior that is accompanied by cortical desynchronization (Crochet and Petersen, 2006; Poulet and Petersen, 2008), was found to suppress the spontaneous activity of L2/3 PCs but cause enhanced firing in a subset of L5 PCs (De Kock and Sakmann, 2009). Whisking-related desynchronization suppressed FS activity while enhancing spiking of non-FSs (Gentet et al., 2010). Application of cholinergic agonists to thalamus under urethane anesthesia also causes a desynchronized state in the barrel cortex in which L2/3 PCs are suppressed; however, in contrast to our results and observations in superficial layers (Gentet et al., 2010), FSs in thalamic recipient layer were strongly enhanced by this manipulation (Hirata and Castro-Alamancos, 2010). In the visual cortex, basal forebrain stimulation causes a decrease in multiunit firing rates in superficial layers, but a mean increase in all others (Goard and Dan, 2009), largely consistent with our results; however, a second study (Niell and Stryker, 2010) found that running in a head-restrained condition (also expected to cause desynchronization) had little effect on spontaneous firing rates, except for a subset of putative FSs which showed a dramatic increase. The differences between these results—such as diverse effects on FSs—may result from either differences in the form of desynchronizing conditions (e.g., drug administration, induced cortical activations, active behaviors), or from differences between cortical areas and/or layers. Nevertheless, a common picture does appear to emerge from these multiple studies, in particular with desynchronization evoking suppression of PC activity in the superficial layers and diverse effects elsewhere, consistent with our results. In addition, although effects of electrical PPT stimulation on downstream structures are likely complex (Steriade et al., 1991; Jones, 2003; Winn, 2006; Schofield et al., 2011), we observed consistent results across different conditions.

It has been suggested that spiking phases (up states) of the synchronized state consist of fragmentary moments of cortical activity similar to wakefulness (Destexhe et al., 2007). Our data indicate at least one way in which this notion is incomplete (see also Ushimaru et al., 2012). Superficial-layer activity during up states is sparse (Sakata and Harris, 2009); however, desynchronized state firing rates are rather lower than synchronized state rates (which also include silent phases of the synchronized state). The mechanisms of this decrease in rate are currently uncertain. It seems improbable that it results from inhibition, at least from fast-spiking cells, as putative FSs also reduce their firing rates during desynchronized epochs. Neuromodulatory systems are active during desynchronization (Pace-Schott and Hobson, 2002; Jones, 2003; Harris and Thiele, 2011), which may hyperpolarize certain classes of neuron (Gulledge and Stuart, 2005; Gulledge et al., 2007). Another possibility is firing rate adaptation. If superficial layer neurons showed particular tendency of spike adaptation, one might expect them to fire strongly in a spiking phase of the synchronized period, but not be able to sustain this activity in prolonged desynchronized state activity. Indeed, superficial PCs show stronger adaptation than at least fast-spiking cells in the auditory cortex (Schiff and Reyes, 2012), and lower PC activity might in turn lead to lower activity of fast-spiking cells.

What function might laminar-dependent changes in firing rates have for information coding? The reduction of spontaneous firing in superficial cells is likely to increase signal-to-noise ratios (SNRs) to encode sensory signals (Livingstone and Hubel, 1981). Since L2/3 population activity is sparse and spatially localized (Sakata and Harris, 2009; Bathellier et al., 2012; Harris, 2012) and sparse coding is in general beneficial to readout signals for downstream networks (Olshausen and Field, 2004), the improvement of SNRs could be further beneficial for their downstream targets. In deep layers, on the other hand, increased baseline firing in a subset of PCs might appear to increase baseline “noise”. However, because this baseline activity has a tonic structure rather than exhibiting alternating up and down phases, desynchronization could instead result in the reduction of output fluctuations from those cells compared to the synchronized state. We therefore suggest that it could allow downstream networks to detect subtle deviations, with the increased baseline activity helping to reach threshold for weak signals as well.

In conclusion, we assessed the effect of cortical states on spontaneous spiking activity across cell-types of the auditory cortex. Spiking activity in superficial PCs and putative FSs is suppressed during desynchronization. We hypothesize that this reduction of internal noise is beneficial for the brain to detect behaviorally relevant stimuli.

### Conflict of interest statement

The authors declare that the research was conducted in the absence of any commercial or financial relationships that could be construed as a potential conflict of interest.
